# Cyclovirobuxine D inhibits hepatocellular carcinoma growth by inducing ferroptosis of hepatocellular carcinoma cells

**DOI:** 10.1007/s12672-024-00940-2

**Published:** 2024-04-02

**Authors:** Xinru Jiang, Hongdan Li, Yang Liu

**Affiliations:** 1 The Third Affiliated Hospital of Jinzhou Medical University, Section 5, No.2, Heping Road, Jinzhou, 121000 Liaoning China; 2 Life Science Institute, Jinzhou Medical University, Section 3, No.40, Songpo Road, Jinzhou, 121000 Liaoning China; 3 Department of Clinical Laboratory, The Third Affiliated Hospital of Jinzhou Medical University, Section 5, No.2, Heping Road, Jinzhou, 121000 Liaoning China

**Keywords:** Hepatocellular carcinoma, Cyclovirobuxine D, Ferroptosis, Lipid peroxidation

## Abstract

**Objective:**

Hepatocellular carcinoma (HCC) is one cancer with high death rates. Nowadays, there are no effective drugs to treat it. Cyclovirobuxine D (CVB-D) is the primary ingredient of the traditional Chinese medicine (TCM) *Buxus microphylla*. Here, we try to explore the impacts of CVB-D on human HCC cells and explain the potential mechanisms.

**Methods:**

HepG2 and Huh-7 cells were used for our experiments. The cell viability and half inhibitory concentration (IC50) were detected by MTT assays. The apoptosis ratio was examined by Annexin V-FITC/7AAD staining and flow cytometry (FCM). The Fe^2+^ content was examined by ferrous ion content assays. The malondialdehyde (MDA) content was evaluated by lipid peroxidation MDA assays. The reactive oxygen species (ROS) level was examined by the DCFH-DA probe. The expression of apoptotic markers (Bax and Bcl-2) and ferroptosis-related proteins (GPX4 and FSP1) was detected by western blotting. The in vivo curative effect of CVB was explored using xenograft models established in C-NKG mice.

**Results:**

The cell viability could be inhibited by CVB-D in HepG2 and Huh-7 cells. The IC50 value of CVB-D on HepG2 and Huh-7 cells are 91.19 and 96.29 µM at 48 h, and 65.60 and 72.80 µM at 72 h. FCM showed that the apoptosis rate was increased by CVB-D in HepG2 and Huh-7 cells. Next, ferrous ion content assays showed that the level of Fe^2+^ was increased by CVB-D in HepG2 and Huh-7 cells. Then, we found the level of MDA and ROS was increased by CVB-D. And the Fe^2+^ promotion by CVB-D could be reversed by Fer-1. Additionally, western blotting assays showed that the expression of GPX4 and FSP1 was inhibited by CVB-D in HepG2 and Huh-7 cells. Moreover, in vivo, CVB-D displayed excellent anticancer effects in HCC tumor-bearing C-NKG mice.

**Conclusion:**

CVB-D suppresses the growth in HCC cells through ferroptosis.

## Introduction

Predominantly hepatocellular carcinoma (HCC), liver cancer is the sixth most often diagnosed cancer and the third major cause of cancer mortality globally [1, 2]. HCC is closely associated with viral hepatitis, nonalcoholic steatohepatitis, alcoholic hepatitis, cirrhosis, and aflatoxin contamination [3]. Recent years have seen tremendous progress in the early detection and management of HCC, which has decreased the disease’s death and morbidity rates [4]. However, many nations and regions, including China, still face high death rates [3]. The World Health Organization projects that liver cancer will claim over 1 million lives by 2030 [5]. Therefore, the identification of potent medications for the treatment of liver cancer is urgently needed.

Traditional Chinese medicine (TCM) has been utilized in China for many thousands of years to prevent liver cancer and has also been shown to be effective in treating liver cancer in modern China [6]. An alkaloid component called cyclovirobuxine D (CVB-D) is extracted from the root of the *Buxus microphylla*. *Buxus microphylla* has been used to treat/prevent numerous cardiovascular ailments in China for centuries [7, 8]. The ability of CVB-D to cause autophagic cell death in human breast cancer cells was initially discovered by Lu et al. in 2014 [9]. CVB-D was later shown by Zhang et al. to promote apoptosis and stop the cell cycle in the G2 phase, thus inhibiting the growth of HCC cells [10].

As a novel cell death mechanism, ferroptosis has great potential for clinical application in cancer therapy [11]. Unlike apoptosis, different types of necrosis, and autophagy, ferroptosis is a type of cell death caused by iron-dependent lipid peroxidation and excessive production of reactive oxygen species (ROS). Its main cytological features include diminished or absent mitochondrial cristae and ruptured outer mitochondrial membrane [12–14]. There is clear evidence that ferroptosis has an antitumor impact in experimental tumor models [15]. In addition, ferroptosis management can overcome immunotherapy, targeted treatment, and conventional chemotherapy drug resistance [16]. The primary regulating mechanisms of ferroptosis are iron, lipid peroxidation, and amino acid metabolism. The abundance of free iron and high level of ROS in tumor cells give a theoretical justification for the therapeutic use of ferroptosis in the therapy of cancers [17]. So far, CVB-D could induce autophagy, apoptosis, mitochondrial damage, and mitophagy in many cancers. However, there is no research describing the effects of CVB-D on ferroptosis.

In this study, we will try to explore the biological activities of CVB-D on HCC cells and explain the potential mechanisms of CVB-D on ferroptosis.

## Material and methods

### Cell culture

The two human HCC cell lines Huh7 and HepG2 were obtained from Life Science Institute, Jinzhou Medical University and the origin of the cell lines were verified by STR profiling. Cells were maintained with Dulbecco’s modified Eagle’s medium (DMEM) (Gibco, Thermo Fisher Scientific, China) containing 10% fetal bovine serum (FBS) (Gibco, Thermo Fisher Scientific, China), 100 U/ml penicillin (Procell, China), and 100 mg/ml streptomycin (Procell, China) in an incubator (Thermo Fisher Scientific, Waltham, MA, USA) at 37℃ with 5% CO2.

### Cell viability

CVB-D (C117989, Aladdin, China) was dissolved in methanol to create a 20 mM stock solution. HepG2 and Huh7 cells were maintained in 96-well plates and exposed to different doses of CVB-D (0–120 µM) for 48 or 72 h. The formazan crystals in the cells were dissolved with DMSO following a 4-h incubation in a full medium supplemented with MTT. The absorbance was measured by a microplate reader at 490 nm.

### Annexin/7-AAD staining

HepG2 and Huh7 cells were grown on 6-well plates and treated with various CVB-D (0–100 µM) for 48 h. The cells were digested and washed in the dark for Annexin V-FITC and 7-AAD staining (K1139, APExBIO, USA). The cells were detected by flow cytometry (FCM).

### Ferrous ion content assay

A total of five million cells were collected and suspended in 1 ml extraction solution. The cells were disrupted using ultrasonic waves and followed by centrifugation at 4 °C for 10 min at a speed of 10,000–12,000 g. The resulting supernatant was obtained and was supplemented with ferrous ion detection reagent (BL1147A, Biosharp, China). The mixture was thoroughly mixed and placed at room temperature for 15 min. The absorbance was measured by a microplate reader at 562 nm.

### Lipid peroxidation MDA assay

A total of one million cells were collected and suspended in 100 µl extraction solution. The cells were then disrupted using ultrasonic waves and followed by centrifugation at 4 °C for 10 min at a speed of 10,000–12,000 g. The resulting supernatant was obtained and was supplemented with the MDA detection reagent (BL904, Biosharp, China). The mixture was thoroughly mixed and was incubated at 100 °C for 15 min. Subsequently, the mixture was cooled in a water bath to room temperature and centrifuged at 1000 g for 10 min at room temperature. The resulting supernatant was transferred into a well of a 96-well plate, followed by measuring the absorbance at 532 nm using an enzyme-labeled analyzer. The contents of MDA were evaluated by comparing with the standard curve of MDA.

### Reactive oxygen species level

DCFH-DA staining (BB4705, BestBio, China) was used to evaluate the intracellular ROS level. HepG2 and Huh7 cells were maintained in 12-well plates. After treatment, the cells underwent three rounds of PBS washing before being exposed to 10 µM DCFH-DA for 30 min at 37 °C. After one more PBS washing, the fluorescence of the cells was observed at 485 nm excitation (535 nm emission) using a fluorescence-inverted microscope.

### Western blotting

Target proteins were isolated from cells and identified by western blotting at the cellular level. The bicinchoninic acid assay (BCA) protein assay kit (P0009, Beyotime, China) was used to measure the protein quantities in the samples. Next, sodium dodecyl sulfate–polyacrylamide gel electrophoresis was used to separate 20–30 mg of each sample’s soluble proteins, which were then electrophoretically transferred onto polyvinylidene difluoride (PVDF) membranes. The primary antibodies were diluted into 5% nonfat milk as follows: Bax antibody (ab32503, Abcam, UK, 1:5000), Bcl-2 antibody (WL01556, Wanleibio, China, 1:500), GPX4 antibody (ab125066, Abcam, UK, 1:1000), FSP1 antibody (342551, Zenbio, China, 1:1000), and GAPDH antibody (ab137959, Absin, China, 1:5000). Membranes were incubated with primary antibodies at 4 °C overnight after being blocked with 5% nonfat milk for 1 h at room temperature. Following three rounds of washing in Tris-buffered saline (TBS) containing 0.1% Triton, the membranes were then subjected to horseradish peroxidase (HRP)-conjugated secondary antibody (CST, USA, 1:10,000) for 1 h at room temperature. The protein bands were then seen using Clarity™ Western ECL substrate (BL520B, Biosharp, China), and ImageJ was used to do the quantification analysis.

### Anti-HCC effect of CVB-D in vivo

Subcutaneous tumor models of hepatocellular carcinoma (HCC) were established in 4-week-old male C-NKG mice obtained from Jiangsu, China. In this study, two million HepG2 and Huh-7 cells were injected suspended in 200 mL PBS into the right dorsal subcutaneous area of C-NKG mice. After two weeks, mice with tumors measuring approximately 50mm^3^ in volume were randomly assigned to either the control group or the CVB-D treatment group (10 mg/kg, intraperitoneal injection, every 2 days). Each group consisted of five mice, and the treatment schedule was followed accordingly.

### Statistical analysis

Software called GraphPad Prism 9.5 was utilized to analyze the findings. A one-way analysis of variance (ANOVA) was used to assess differences between the control and experimental groups. The data were given as mean ± standard deviation (n = 3).

## Results

### Cytotoxicity of CVB-D to HCC cells

**Fig.1:**
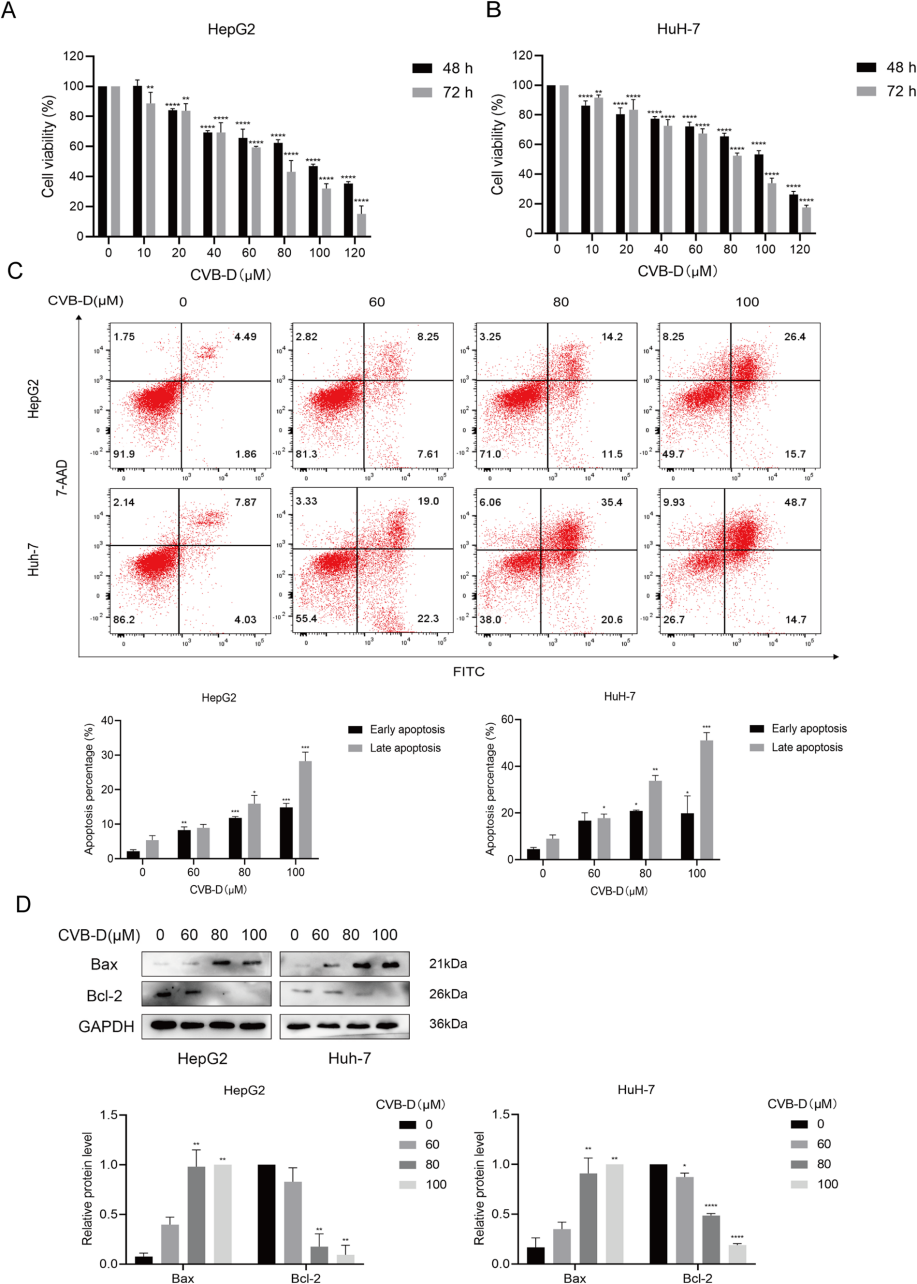
CVB-D influenced the cell viability of HepG2 and Huh-7 cells. **A**, **B** HepG2 and Huh-7 cells were exposed to different doses of CVB-D (0–120 µM) for 48 and 72 h, and the cell viability was measured by MTT assays; **C** Effect of CVB-D on the apoptosis of HCC cells was detected by Annexin V-FITC/7-AAD staining and the percentage of early and late apoptotic cells after treatment with various concentrations of CVB-D;** D** The protein expression of Bax and Bcl-2 was detected by western blotting. The amount of protein in term of the band intensity was analyzed by ImageJ. *P < 0.05, **P < 0.01, ***P < 0.001, ****P < 0.0001 vs. control group

To evaluate the impact of CVB-D on HepG2 and Huh7 cells, the cytotoxicity of CVB-D on HCC cells was measured by MTT assays. The result showed that the cell viability of HCC cells was significantly decreased. Furthermore, this suppression was strongly correlated with the concentration and duration of CVB-D exposure (Fig. 1A, B). The IC50 value of CVB-D on HepG2 and Huh-7 cells are 91.19 and 96.29 µM at 48 h, and 65.60 and 72.80 µM at 72 h.

After treating HCC cells with CVB-D, the apoptosis ratio was examined by Annexin V-FITC/7-AAD staining. The findings demonstrated that after CVB-D treatment, the apoptosis ratio of HepG2 and Huh-7 cells increased significantly (Fig. 1C). The apoptosis ratio of HepG2 under 0, 60, 80, and 100 µM CVB-D treatment are 6.35%, 15.86%, 25.7%, and 42.1%. The apoptosis rates of Huh-7 under 0, 60, 80, and 100 µM CVB-D treatment are 11.9%, 41.3%, 56%, and 63.4%. Western blot assays showed that the expression of an antiapoptotic m marker (Bcl-2) gradually decreased, whereas that of an apoptotic marker (Bax) gradually increased as the CVB-D concentration increased (Fig. 1D).

### CVB-D increased the iron ion content in HCC cells

**Fig.2:**
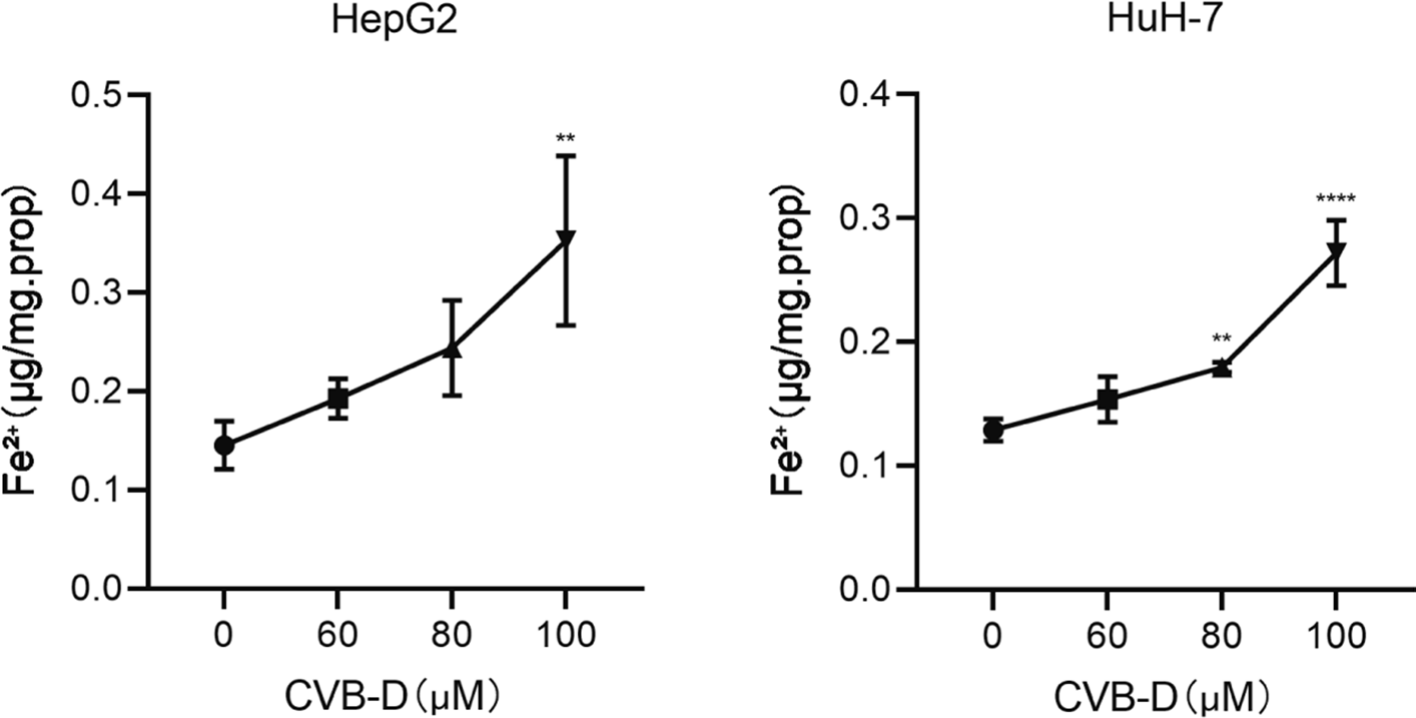
CVB-D promoted the ferrous ion content in HepG2 and Huh-7 cells. HepG2 and Huh-7 cells were exposed to different doses of CVB-D (60,80 and 100 µM) for 48 h. The ferrous ion content was detected by a ferrous ion content assay kit. *P < 0.05, **P < 0.01, ***P < 0.001, ****P < 0.0001 vs. control group

To explain whether the inhibition of CVB-D on HCC is related to ferroptosis, ferrous ion was detected first. The assays showed that the ferrous ion content was promoted by CVB-D in HepG2 and Huh7 cells in a dose-dependent manner. Iron concentration was increased significantly when treated with CVB-D at 100 µM in HepG2 and Huh-7 cells (Fig. 2). It was suggested that ferroptosis might be the mechanism of CVB-D's effect on HCC cells.

### CVB-D induced ferroptosis in HCC cells

**Fig.3:**
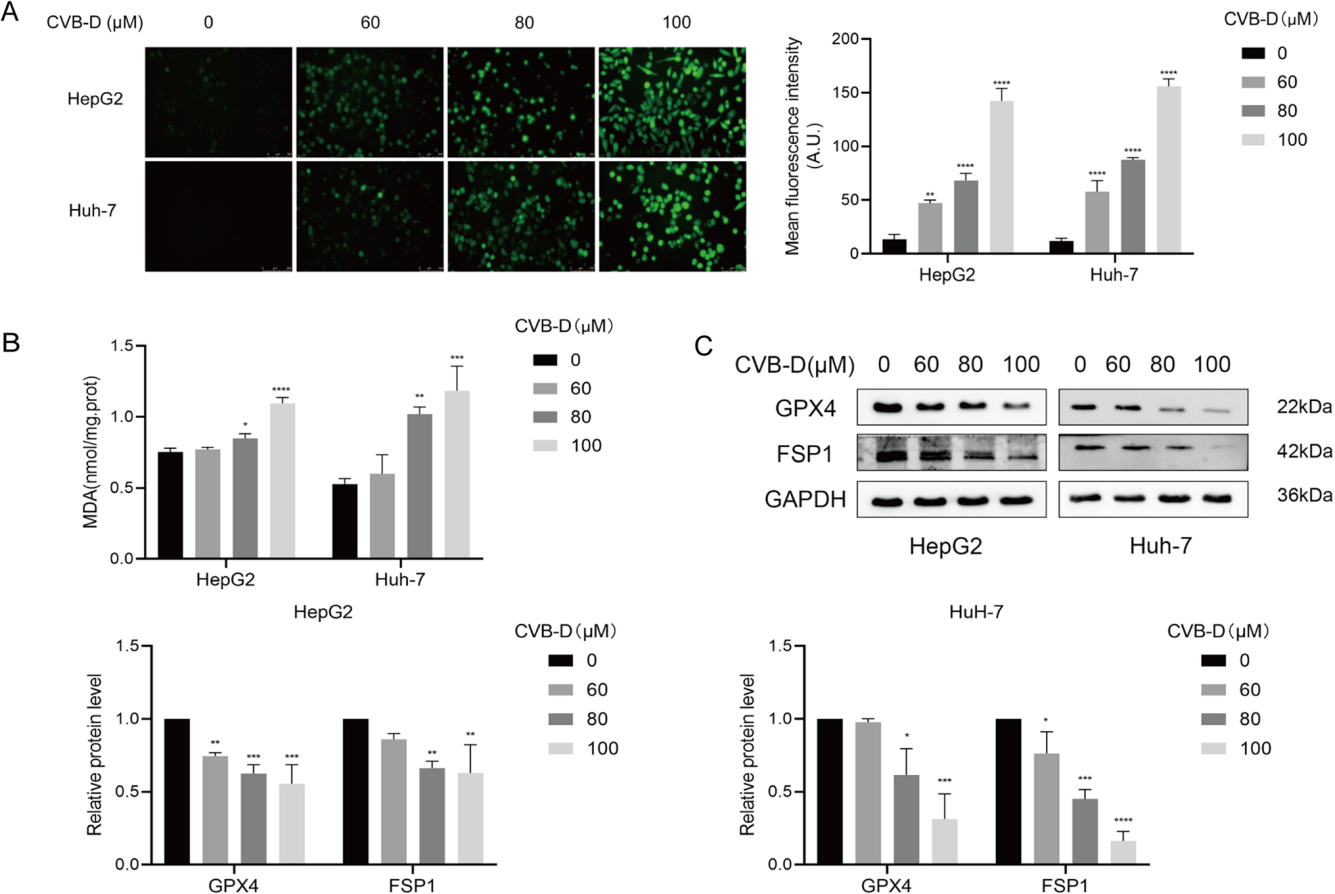
CVB-D induced Ferroptosis in HepG2 and Huh-7 cells. HepG2 and Huh-7 cells were exposed to different doses of CVB-D (60, 80 and 100 µM) for 48 h. **A** The level of ROS was detected by fluorescent probe DCFH-DA. **B** The level of MDA was detected by lipid peroxidation MDA assay kit. **C** The protein expression of GPX4 and FSP1 was detected by western blotting. The amount of protein in term of the band intensity was analyzed by ImageJ. *P < 0.05, **P < 0.01, ***P < 0.001, ****P < 0.0001 vs. control group

For CVB-D to promote the ferrous ion content in HCC, the effect of CVB-D on ferroptosis was further investigated. ROS and MDA assays showed a clear dose-dependent alteration in the level of CVB-D at 0, 60, 80, and 100 µM (Fig. 3A, B). Western blot assays showed that the level of GPX4 and FSP1 was decreased obviously at 100 µM CVB-D and displayed a dose-dependent manner (Fig. 3C). The relative protein level was analyzed by ImageJ. As 100 µM CVB-D could promote ferroptosis in HepG2 and Huh-7 cells, 100 µM CVB-D was selected for further experiments.

Together, the findings demonstrated that ferroptosis plays a role in CVB-D's ability to suppress the growth of HepG2 and Huh-7 cells.

### Inhibiting ferroptosis reversed the effects of CVB-D on HCC cells

**Fig.4:**
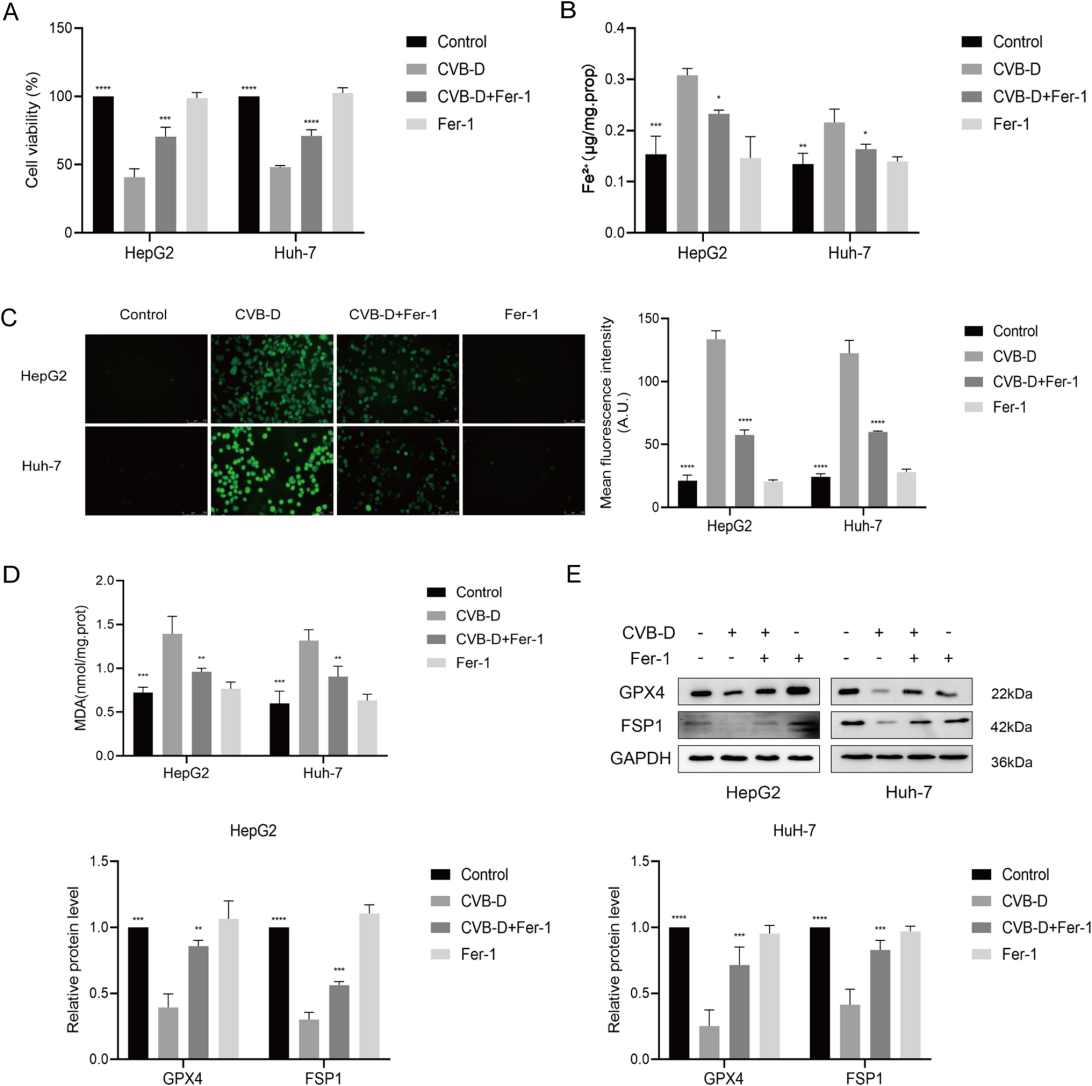
Inhibition of ferroptosis by Fer-1 attenuates cell death induced by CVB-D in HCC cells. HepG2 and Huh-7 cells were treated with or without 100 µM CVB-D and 2 µM Fer-1 for 48 h. **A** The cell viability was measured by MTT assay. **B** The ferrous ion content was detected by a ferrous ion content assay kit. **C** The level of ROS was detected by fluorescent probe DCFH-DA. **D** The level of MDA was detected by lipid peroxidation MDA assay kit. **E** The protein expression of GPX4 and FSP1 was detected by western blotting. The amount of protein in term of the band intensity was analyzed by ImageJ. *P < 0.05, **P < 0.01, ***P < 0.001, ****P < 0.0001 vs. CVB-D group

To explore the contribution of ferroptosis on the anticancer effect of CVB-D more thoroughly, here, Fer-1, a specific ferroptosis inhibitor, was utilized to inhibit ferroptosis in CVB-D-treated cells. MTT assays showed that the cell death induced by CVB-D was rescued by Fer-1 (Fig. 4A). Meanwhile, ferrous ion detection showed that iron accumulation promoted by CVB-D was reversed by Fer-1 (Fig. 4B). ROS assays indicated the expression of ROS in CVB-D treatment pretreatment was decreased with Fer-1. The fluorescence intensity of ROS in the CVB-D + Fer-1 group was stronger than in the CVB-D group (Fig. 4C). Additionally, MDA assays showed that Fer-1 led to a reduction compared with CVB-D single treatment (Fig. 4D). Further, western blotting also revealed that the level of GPX4 and FSP1 in CVB-D treatment was reversed by Fer-1 (Fig. 4E). The relative protein level was analyzed by ImageJ in Fig. 4E.

These findings generally showed that cell death and ferroptosis alterations in HepG2 and Huh-7 cells treated with CVB-D could be prevented by Fer-1.

### CVB-D inhibits tumor growth

**Fig.5:**
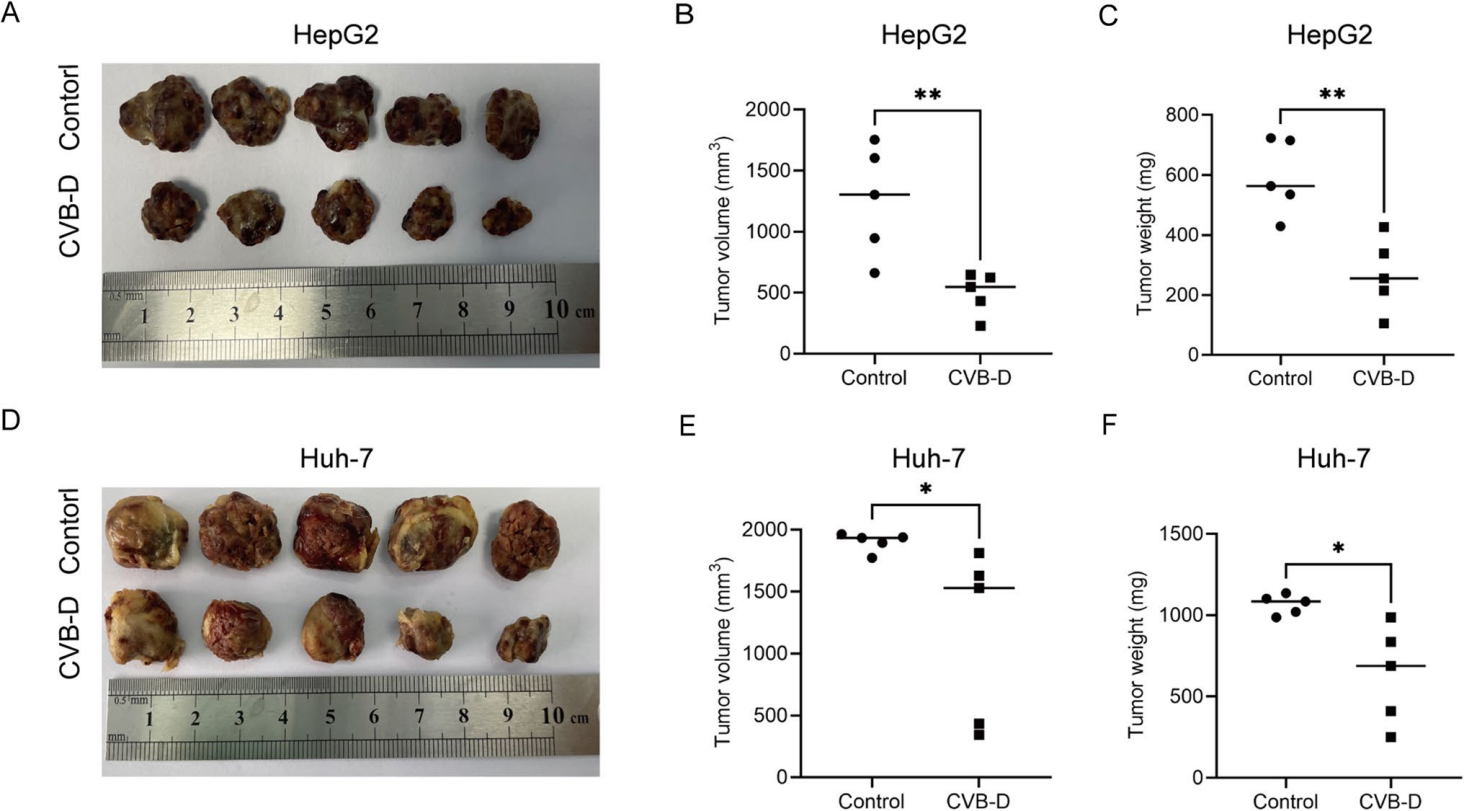
CVB-D suppresses the progression of HCC in vivo. **A**, **D** The image, **B**, **E** weight, and **C**, **F** volume of xenograft tumors detached from the C-NKG mice after 14 days of CVB-D treatment

To investigate the therapeutic effects of CVB-D on HCC in vivo, we conducted anti-HCC experiments with CVB-D in HepG2 and Huh-7 tumor-bearing C-NKG mice. Our study showed that CVB-D treatment significantly decreased tumor growth in vivo, which was further confirmed by the significantly decreased tumor weights and volumes observed in the CVB-D treatment group (Fig. 5). In general, CVB-D exhibits significant anti-liver cancer effects in tumor-bearing mice.

## Discussion

Although clinical therapy has shown significant improvements in recent years, HCC continues to rank among the fatal types of cancer among humans, with a 5-year survival rate below 10 percent [18]. One of the challenges encountered in managing HCC patients pertains to the limited availability of efficacious drugs and the significant incidence of adverse reactions associated with the current therapeutic interventions used in clinical practice [19]. In China, TCM is esteemed as a valuable medical approach and a precious resource. TCM plays a significant role in mitigating the adverse reactions resulting from radiotherapy and chemotherapy. Moreover, it contributes to a longer lifetime and a higher survival rate [20]. As a drug of TCM, CVB-D could induce autophagy, apoptosis, mitochondrial damage, and mitophagy in many cancers. However, there is no research on CVB-D-induced ferroptosis-associated cell death. Recent studies demonstrate the crucial role ferroptosis plays in regulating the growth of malignancies, such as HCC, RCC, and non-small-cell carcinoma (NSCLC) [21, 22].

For the role of CVB-D on ferroptosis is not clear, we try to explore the mechanism of CVB-D on HCC. Firstly, to explore the influence of CVB-D on HCC, we used MTT and AV/PI staining assay. The results demonstrated that CVB-D hindered the growth of HCC cells, and promoted the apoptosis of HCC (Fig. 1A and B). CVB-D could inhibit the growth of HCC. Then, we detected Fe^2+^ concentration and found CVB-D increased cellular Fe^2+^ content in a concentration dependent way (Fig. 2). Lipid peroxidation and iron metabolism have a strong association with the regulation of iron-induced cell death. The central mechanism of iron-driven cell death entails the buildup of products resulting from lipid peroxidation within cells. Iron is deemed an essential factor for the accumulation of lipid peroxides and the initiation of ferroptosis. So, we next detected the expression of ROS and MDA, and the findings showed that CVB-D promoted ROS and MDA in HCC as a concentration dependent manner (Fig. 3). GPX4 is the primary enzyme preventing ferroptosis [23], which prevents ferroptosis by changing lipid hydroperoxides into non-toxic lipid alcohols [24]. It is essential to note that the inhibition or deletion of GPX4 in HCC cells can directly trigger the buildup of lipid peroxides, ultimately leading to ferroptosis [25]. FSP1 is a potent ferroptosis suppressor [26]. In cancers, the crucial expression of FSP1 can predict the effectiveness of iron apoptosis-inducing medications, which function alongside the traditional glutathione-dependent GPX4 pathway as a strong inhibitor of ferroptosis [27].

Our research determined that CVB-D had a significant impact on the iron, MDA, and ROS levels in HCC cells. Furthermore, the protein expression of the anti-ferroptosis genes GPX4 and FSP1 were notably reduced by CVB-D. To further validate our findings, we also conducted the iron, MDA, ROS and WB assay using the ferroptosis inhibitor Fer-1 before CVB-D treatment. The results showed Fer-1weakened the ferroptosis induced by CVB-D in HCC cells (Fig. 4). In vivo, CVB-D displayed excellent anticancer effects in HCC tumor-bearing C-NKG mice (Fig. 5). Our study showed that CVB-D can not only promote apoptosis of liver cancer cells, but also promote ferroptosis of liver cancer cells. Therefore, the combination of ferroptosis-inducing drugs with other anticancer methods can be used to improve the efficacy of anti-tumor therapy.

**Fig.6:**
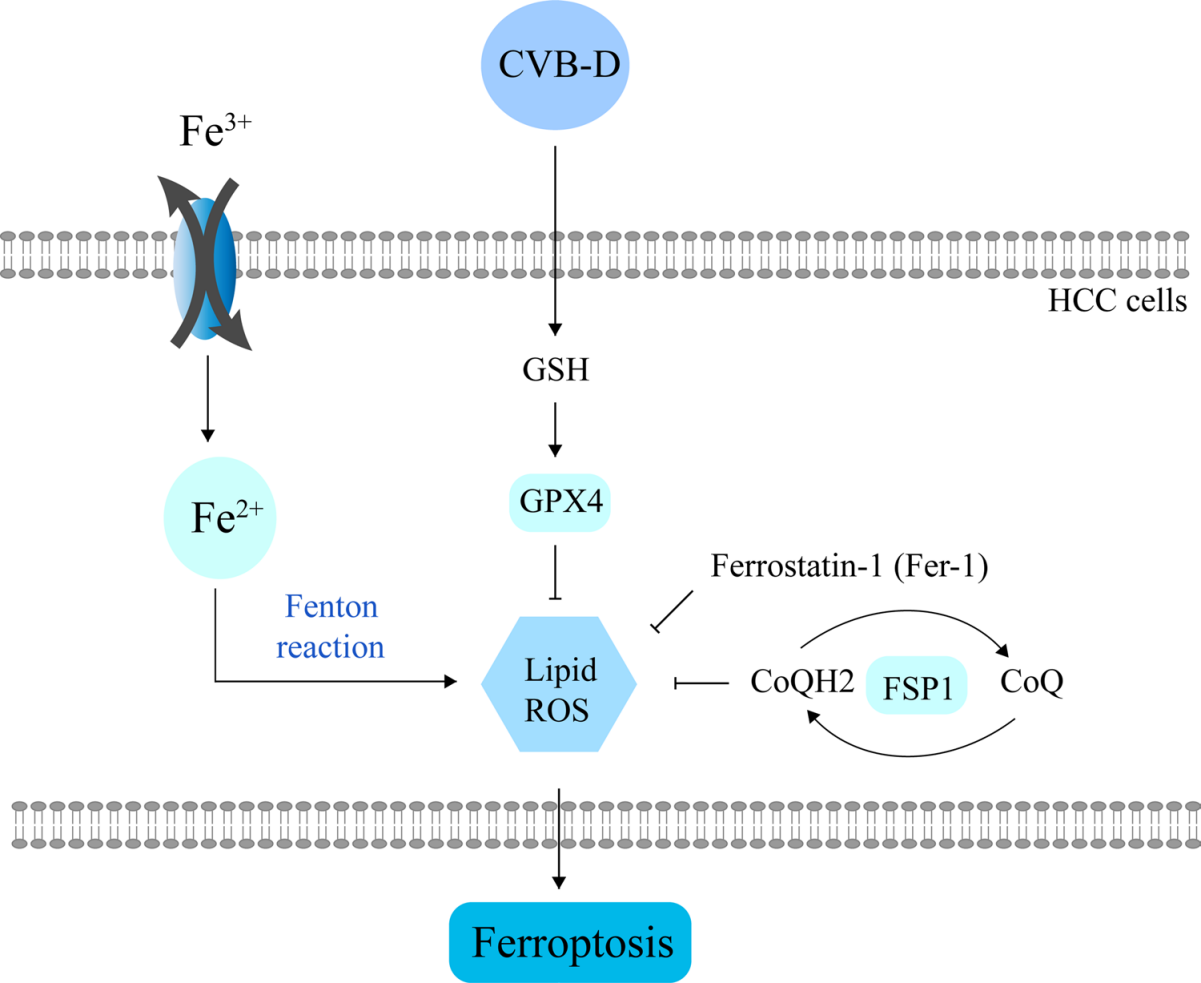
Schematic diagram of CVB-D induced ferroptosis in HCC cells

Together, we show the regulatory mechanism of CVB-D in inducing ferroptosis in the following schematic diagram (Fig. 6).

## Conclusion

CVB-D is an effective drug in HCC therapy and suppresses the growth of cancer cells through ferroptosis.

## Data Availability

The datasets generated and analyzed during the current study are available from the corresponding author on reasonable request.
